# Research on the Construction of English Autonomous Learning Model Based on Computer Network-Assisted Instruction

**DOI:** 10.1155/2022/8646463

**Published:** 2022-06-15

**Authors:** Mijuan Tian, Rong Fu, Qianjun Tang

**Affiliations:** School of Educational Sciences, Leshan Normal University, Leshan, Sichuan 614000, China

## Abstract

As a supplement to traditional teaching methods, computer-assisted teaching methods can reflect modern educational concepts, such as creating student-led, teacher-led environments. The goal of college English education is to enable them to communicate effectively in English in their future academic, work, and social interactions, while also developing students' self-learning skills. Chinese society improves overall cultural competence and adapts to the needs of international communication. Self-directed learning is not static and will increase or decrease with time, discipline, and conditions and is an evolving process. Understanding learning, taking responsibility for one's own learning, and learning how to learn are all beneficial. Students abound in school life and even throughout their lives. In this paper, we try to propose a computer-based method for constructing an independent English learning model based on a practical study of computer network technology for the development of self-learning ability of non-English majors in a university. This paper uses comparative analysis techniques to compare traditional paper-and-pencil examinations and computer-based online evaluations and analyzes the effects of each. The survey showed that 81% of the students preferred the computer-based assessment. Therefore, the focus of this research is to strengthen the oral English training in college and create an authentic English learning environment for students to really feel the standard English pronunciation, intonation, and knowledge of grammar, listening, reading, writing, and translation.

## 1. Introduction

In the context of the rapid growth in the number of college students in China and the relatively limited educational resources, the existing single-class teaching model was improved and a computer and new teaching model was developed [1]. With the support of modern information technology, especially network technology, English education is developing in the direction of personalized learning and self-study which is not limited by time and place [2]. Traditional teaching methods centered on teachers, classrooms, and books can no longer meet the needs of the information society [3]. Modern educational technology, represented by network-based computer-assisted education, is having a profound impact on education [4]. At the same time, all kinds of latest information on the Internet and constantly updated educational models are affecting traditional education, and both students and teachers are overwhelmed by rich educational resources [5].

In the era of knowledge and information explosion, individual learning ability is a key factor for sustainable development [6]. Assistive teaching methods such as computers play an important role in teaching English listening, reading, writing, and translation [7]. Teachers teach English through multimedia and other supplementary teaching resources. Teaching content is presented in a three-dimensional combination of images, sound, animation, and text, which changes the traditional teaching methods and significantly improves teaching efficiency [8]. Allowing learners to learn independently through the Internet has become an important means to cultivate students' comprehensive quality [9]. The quality of students has also changed significantly, with students becoming more individualized and the traditional elite education not interconnecting with the needs of students [10]. In the context of web-assisted teaching, teachers and students inevitably fall into the confusing stage of “how to teach and how to learn” [11]. This has put forward higher demands on the audiovisual learning and teaching of English for college students. The independent learning mode of English based on computer network-assisted teaching allows students to learn anytime and anywhere, choose the materials that suit their needs, and should be able to complete listening and speaking training that cannot be done in traditional classrooms, including recording, comprehension, and student learning. It can test the teacher's teaching and learning at any time, urge students to study actively, and quickly improve practical skills such as English listening and speaking ability.

Creating a computer-assisted teaching system and using the system for educational services can save time for after-school tutors [12]. It can also help students to solve problems in the process of learning computer courses, and the theoretical base tests in the assistive teaching system can help students to integrate the learning effect of the classroom [13]. At a deeper level, teachers and researchers are finding and applying various new pedagogical concepts for teaching university English courses under informational conditions, which significantly increases the efficiency of informational-based physical devices [4]. Computer-supported college English education can apply modern technology to education, realize the perfect combination of information technology and classroom education, effectively improve college English education, and play a positive role in promoting the reform of college English education [14]. Among them, expert system is a model of artificial intelligence, which has successfully penetrated into various fields from theoretical research to practical application, from general thinking to professional knowledge application, and has created great social and economic benefits.

The innovations of this paper are as follows:

1. This system combines teaching evaluation with autonomous learning, strengthens the contact and interaction between teachers and students, and better realizes the purpose of assisting teaching.
2. Using this model, learners can no longer study completely according to the traditional teaching mode and can arrange their own study flexibly and independently, which can be said to be a complete change of the traditional learning form.
3. The system uses an expert system to analyze all students in a specific group (e.g., multiple classes, universities, etc.), allowing the system to provide better education.

The research framework of this paper consists of five parts, which are arranged as follows:

The first part of this paper introduces the research background and significance, and then introduces the main work of this paper. The second part introduces the work related to English autonomous learning mode and computer network-assisted teaching. The third part combs the main construction steps of the expert system and the knowledge representation methods of English autonomous learning model, so that the readers of this paper can have a more comprehensive understanding of the construction of autonomous learning model. The fourth part is the core of the paper. It describes the analysis of students' autonomous learning system from two aspects: BP neural network and implicit knowledge base analysis based on computer network. The last part of the paper is the work summary of the full text.

## 2. Related Work

### 2.1. English Autonomous Learning Model

Using the Internet to acquire knowledge has become an important means of human learning. It allows students who want to learn anytime and anywhere to improve learning efficiency and reduce learning costs. Interactive computer-aided teaching system provides a platform for teachers and students to interact and communicate, facilitates teachers' course instruction, and improves the efficiency of problem solving. It provides a whole new level of modern education and promotes a major new leap in education technology, education system, and education methods. Therefore, how to develop information technology and how to use it more effectively in education has become a matter of concern.

At the same time, Cassady et al. have done a lot of empirical research in combination with theoretical research and developed and built a number of self-learning application centers or platforms, with good results [15]. Mandad et al. propose an integrated technique designed to improve their self-directed learning and self-confidence, and to develop cultural literacy for social development and international exchange [16]. Alotumi adopts network transmission mode, all data are provided through the existing network, and educational content is provided in the form of web page [17]. Chunlin argues that modern information technology should provide new ways of teaching and learning, so that English teaching and learning can move toward autonomy [18]. Lolita et al. have built a computer-assisted instruction system by using the computer laboratory teaching network to establish an environment where students can actively participate in learning [19]. As a comprehensive technology that uses computer as a medium and realizes teaching activities through computer programs, it occupies an important position in the educational circles at home and abroad.

It is necessary to fully apply modern educational technology to college English professors and self-study teachers to further personalize the English teaching model so that all national reform projects can be carried out successfully in an information-based environment.

### 2.2. Computer Network-Assisted Instruction

The history of computer-assisted English education is divided into behaviorism and communicative constructivism. The basic theory of computer-assisted English teaching is the cornerstone of further research on computer-assisted English teaching based on constructivism. Internet provides a large number of shared learning resources for students, which not only meets their English learning needs, but also breaks the boundaries of English teaching time and space, and helps to improve the overall level of English education.

Gao and Jin used artificial intelligence-driven natural language processing techniques to extract, compute, and predict features of articles, which can be classified according to their grammar, usage, structure, style, composition, development, lexical complexity, and inter-word relationships [20]. Aghajani and Amnzadeh developed a new “information modeling” approach. Candidate models are provided by a computer, while emotional selection and human-computer cooperation are performed by a human, so that complex learning can effectively construct satisfactory information models [21]. He provided several supporting features for teacher education and student writing, such as a rich library of configurable questions, exercises, student profile tracking and management, and writing ability diagnosis [22]. As a study of theoretical approaches, Djordjevic and Blagojevic proposed the theory of intelligent transformation of information knowledge, full information theory, and general logic theory [23]. Xie introduced the concept of personalization into traditional computer education systems, enabling the system to teach students according to their cognitive abilities, interest characteristics, etc. [24].

Computer network-assisted training mode allows teachers to create online courses on the platform, while learners choose courses to study independently and learn course content independently. Informatization talents can ensure the normal operation of social order by making maximum use of educational informatization resources, and establishing and improving informatization laws and regulations and informatization policies. Therefore, it is necessary to further study some new technologies that can be applied to college English teaching in order to further promote the reform of College English teaching.

## 3. Construction of English Autonomous Learning Model Based on Network Teaching

### 3.1. Main Construction Steps of Expert System

Learners learn independently through the use of web technologies and web-based learning resources, and instructors create learning contexts and build web-based platforms to accomplish the corresponding teaching tasks [25]. Fuzzy assessment and fuzzy reasoning are good methods for assessing students because there are no clear criteria for assessing their behavior. The weighting factor *w*_*i*_(*i*=1,2,…, *n*) is the weighting coefficient of the sub-condition *A*_*i*_, its value must be given by domain experts, and it satisfies the normalization condition:

(1) $$\begin{array}{c} \sum_{i=1}^{n}W_{i}=1. \end{array}$$

**Figure 1:**
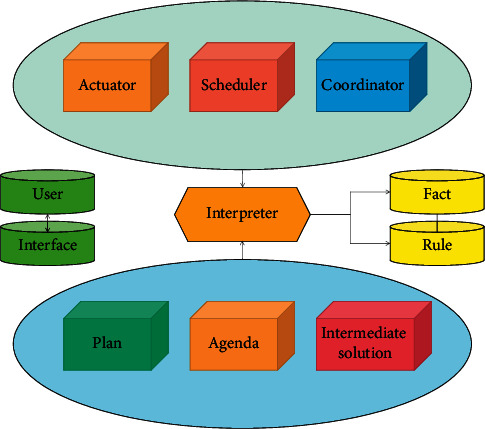
Structure of expert system.

Experts systematically promote educational reform, expand subject knowledge, strengthen skill training, and enlighten and promote learners' thinking. Their functions not only relate to their own value, but also directly affect their position and effect in the educational process. The expert system structure is shown in Figure 1.

First, make a detailed investigation and careful analysis of user needs, and then focus on the application problems to be solved. Task stage is a process in which learners clarify the content of new learning tasks and analyze their relevant conditions and factors according to their existing knowledge system. Let the credibility of each sub-condition *A*_*i*_ be CF(*A*_*i*_), and the credibility of combined evidence is calculated by the following formula:

(2) $$\begin{array}{c} \text{CF}\left(A_{i}\right)=\sum_{i=1}^{n}w_{i}\times \text{CF}\left(A_{i}\right). \end{array}$$

In the computer course-assisted teaching system, students will be able to log in to the system through a browser and register as users to perform corresponding operations, such as browsing course-related information, submitting questions and checking the status of answers through the web browser, taking online tests and viewing statistics, and viewing and submitting assignments. For the *P* th sample, the output error *E*_*P*_ of the network is

(3) $$\begin{array}{c} E_{P}=\frac{1}{2}{\sum_{i=0}^{n-1}\left(tP_{j}-o_{P_{j}}^{\left(l\right)}\right)}^{2}. \end{array}$$

Using a Web browser as the integrated client, integrating the core and functionality of the system into the server and implementing it is the core of the model, and if a Web browser is installed, the client can use it. Assuming that the cost of adding a WME is the average value of the WME added to each *α* memory, there are

(4) $$\begin{array}{c} M_{n}=\frac{1}{n+1}\sum_{m=0}^{n}M_{m,n}. \end{array}$$

**Figure 2:**
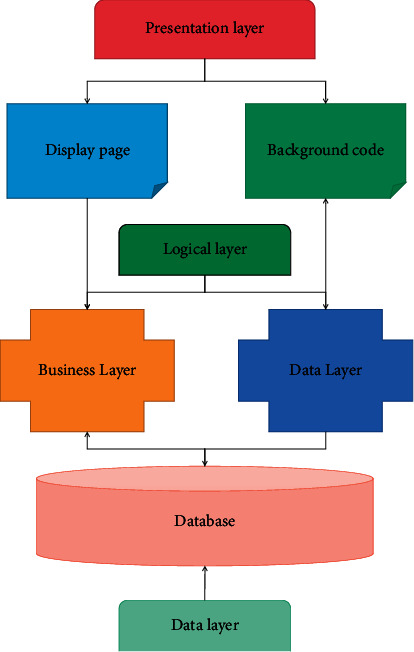
Three-layer structure system.

It is a method of constructing and simulating display system by using object-oriented information modeling concepts, such as classes, relationships, attributes, encapsulation, inheritance, polymorphism, and other mechanisms. The B/S structure achieves powerful functions that previously required complex dedicated software, reduces development costs, and builds a new software system [26]. This structure has become the preferred architecture of reference software today. The three-tier structure system is shown in Figure 2.

Secondly, under the current technological conditions, a large amount of domestic and foreign materials are consulted to collect, summarize, and organize domain knowledge, and appropriate methods are selected for knowledge acquisition, such as manual knowledge acquisition, semi-automatic knowledge acquisition, and fully automatic knowledge acquisition. The goal-setting and planning stage is the process in which learners determine learning goals and learning plans and use learning strategies according to the teaching requirements and standards [27]. The processed result data are then transmitted to the client side, and finally, the client completes the processing and display of the data. The administrator and teacher can manage the module in the background, and the server runs the relevant program module to process the request after receiving it. This protects the data of the object from malicious or unintentional modification by other objects, provides the underlying guarantee for data security, and allows the module to be extended when the requirements change. Therefore, the overhead of deleting a WME is one more than the overhead of adding a WME to find the *β* memory. And the overhead of finding *β* memory at this point is

(5) $$\begin{array}{c} R_{m,n}=\sum_{i=m}^{n}a\circ \prod_{j=1}^{i}a_{j}p_{j}\left(a_{m}=1\right). \end{array}$$

Finally, collect and reorganize the formalized knowledge, including the separation of knowledge base and fact base, knowledge representation, the realization of reasoning mechanism, and man-machine interaction, so as to make it consistent with the characteristics of the problem requiring solution. The strategy execution stage is a process in which learners learn and produce results according to the selected learning strategies [28]. It starts from the teaching objectives and flexibly designs and adjusts the teaching scheme according to the situation of different students. It is a kind of simulation of teachers' computer behavior. According to the requirements of teaching objectives and combined with the cognitive level of learners, it dynamically selects appropriate teaching contents from the Teaching Library. When using the object-oriented method to design a software system, we must first distinguish the types of things in the real world, analyze their properties and functions, and then abstract them into entities and classes that are meaningful in the computer virtual world.

### 3.2. Knowledge Representation of English Autonomous Learning Model

In an expert system, knowledge representation refers to the strategy of representing human knowledge into data structures and system control structures that can be processed by machines. With the use of computer and network in English teaching, students can learn independently and individually according to their interests, needs, tasks, and cognitive styles, thus breaking through the time and space limitations of traditional teaching mode and building an infinite and open teaching space. In the case that the nodes of the hidden layer can be set freely according to the need, the three-layer forward neural network can realize any continuous function with any approximation, and let the output of the layer neuron from the layer *l* neuron *j* to the layer *l*+1 neuron under *P* samples be

(6) $$\begin{array}{c} f\left(x\right)=\frac{1}{1+e^{\left(-x\right)}}. \end{array}$$

**Figure 3:**
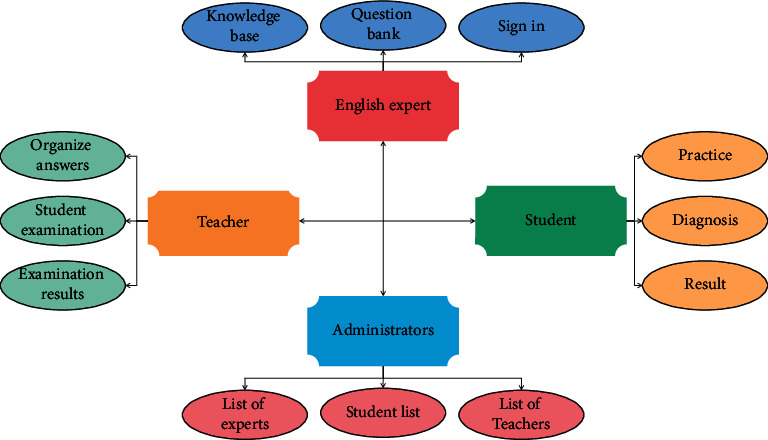
Data flow design.

Knowledge representation is student-centered, and teachers are teaching designers and instructors. It refers to that teachers send the produced teaching videos to students by using micro-classes before class, so that students can carry out extracurricular self-study and master the classroom content in advance. The data flow design of knowledge representation method is shown in Figure 3.

First of all, in the process of building and using an expert system, it is necessary to continuously expand and improve the knowledge in the knowledge base, and whether the knowledge representation is easy to modify and expand knowledge is directly related to the success of the expert system. In the goal-setting stage, learners analyze the learning task, judge their own abilities, envision the learning outcome, and then determine the learning goal based on the combination of these aspects [29]. The number of nodes in the hidden layer of the three-layer network is not chosen arbitrarily, in this system, the number of nodes in the input and output layers can be determined, and the number of nodes in the hidden layer *l* can be given according to the empirical formula.

(7) $$\begin{array}{c} l=\sqrt{m+n}+a,\;a\in \left\{1,2,\ldots ,10\right\}. \end{array}$$

As far as the full review is concerned, the breadth of memory is infinite and forgetting is relatively slow. So, modeling the forgetting law of memory, the Ebbinghaus forgetting curve can be obtained as

(8) $$\begin{array}{c} S\left(t\right)=\frac{1}{1+V_{t}}. \end{array}$$

From the perspective of administrators, teachers, and students, dynamic web technologies are used to transfer information between the client, server, and database for login, logout, browsing, viewing, searching, and testing, as well as for backend management of teachers and administrators. The administrator can manage the teacher user information through this module, and teacher users can access the system backend through the teacher management login screen after the administrator adds teacher user information. In addition to studying the representation of uncertain and imprecise knowledge, imprecise reasoning methods are also explored. Compute the output of each neuron in the implicit layer and output layer of the network:

(9) $$\begin{array}{c} O_{Pj}^{l}=fi\left(\sum_{j}w_{ji}^{l}O_{i}^{l-1}-\theta_{j}^{1}\right). \end{array}$$

Secondly, using concise and consistent knowledge representation methods, the knowledge represented by complex representation methods is difficult to understand. The function of students' personality reasoning is to evaluate students' learning effect, diagnose problems in learning, and evaluate their cognitive ability through students' learning process. Students are able to easily request learning services from the web, and the entire service delivery process requires no human intervention. The formulas for correction weights and thresholds are as follows:

(10) $$\begin{array}{c} w_{ji}^{l}\left(n+1\right)=w_{ji}^{l}\left(n\right)+\eta \delta_{Pj}^{l}o_{Pi}^{1-l}\_\partial \left(w_{ji}^{l}\left(n\right)-w_{ji}^{l}\left(n-1\right)\right). \end{array}$$

Aided review systems are a combination of automatic review and manual review by the teacher, which is done through online annotations, where the teacher selects the original essay with errors and adds tips and suggestions for improvement. In current repository construction practice, the construction of a large repository often involves the integration of several smaller repositories or even fragmented resources.

Finally, the representation should be clear and unambiguous. A clear and unambiguous knowledge representation helps experts understand and directly debug the knowledge in the expert system. The knowledge representation provides code reusability, so that previously designed classes with similar functionality can be masked and expanded in the inherited subclasses without changing the original code, thus making the original code largely reusable. After the integration is completed, the resource base will become a large network with a complex topology. The English language learners who use this university English teaching resource library are likely to face an extremely complex user interface.

## 4. Systematic Analysis of Students' Autonomous Learning

### 4.1. BP Neural Network Analysis

**Figure 4:**
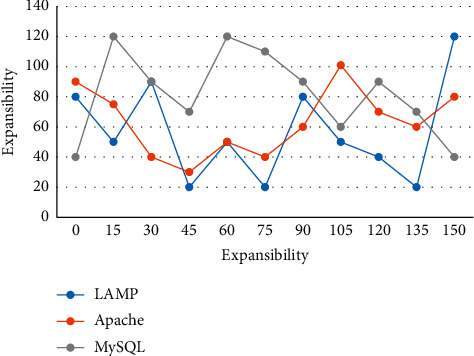
Comparison of two main platforms of web development.

Judging the mastery of students' knowledge points is actually the fault diagnosis of students' knowledge points. The most used and effective in the field of fault diagnosis is the forward multilayer network. Because this kind of network adopts BP algorithm in the process of learning and training, this network is also called BP network. At the same time, there are still some problems in autonomous learning under the network environment, such as backward concept, ignoring design, not focusing on effect, single content, and poor sharing. Therefore, these problems are fully considered in the construction of pattern design. Lamp is a combination of LAMP is a combination of MySQL, Apache, and Linux open-source software, commonly used to build dynamic websites or servers, often used together and highly compatible with each other. Together, they therefore form a powerful web application platform. A detailed comparison of the three is shown in Figure 4.

**Figure 5:**
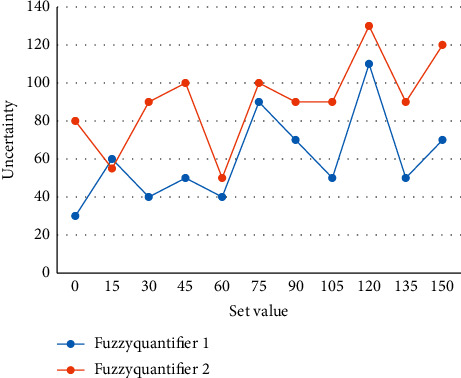
Uncertainty comparison of different quantifiers.

First, the adjacent layers in the network are connected by interconnection, there is no connection between neurons in the same layer, and there is no direct connection between the output layer and the input layer. It is a mode of operation in which the server receives requests from the browser, then gets the data from the database, runs the processing on the server, and returns the results to the browser. When the student user registers, the information entered by the user will be verified by setting the verification from operation, such as QQ number can only be in numeric format and special format of e-mail address. The information entered by the student user can only be written to the database if it passes the verification; otherwise, the system will give a prompt and need to re-enter. When generating random numbers, the system automatically compares the newly generated random numbers with the existing random numbers, and if it is found to be equal to the existing random numbers, the newly generated random numbers will be discarded and generated again to ensure that all random numbers are not the same. The fuzzy output variables of the system have the reliability of the conclusion and the confidence level is propagated sequentially.  Figure 5 shows the uncertainty of the following two fuzzy quantifiers.

Secondly, the number of samples required for network training depends on the complexity of the input-output nonlinear mapping relationship; the more complex the mapping relationship is, the more noise the samples contain, the larger the number of samples required to ensure a certain mapping accuracy, and the larger the network size. The core part of the system function is concentrated on the server, and the main application part is concentrated on the client with web browser, which makes the development and maintenance of the system simpler. The task of the teaching strategy reasoner is to select appropriate learning resources and teaching methods for students through corresponding reasoning algorithms based on the personalized information provided by the student model. Through the theoretical self-test and data statistics module in the system, students can take online tests after learning the theoretical knowledge, through which they can know their knowledge mastery, and after the tests, they can record the results and view the results and histogram, so that they can understand their learning situation in time. Therefore, the independent learning platform must be an Internet-based system that makes full use of computer technology to make the learning content rich and vivid, and is designed to be modularized for easy maintenance and expansion in the future. The database is designed to record the test results of students, and the design is shown in Table 1.

Finally, under the condition that the hidden layer nodes can be freely set as required, any continuous function with arbitrary approximation can be realized by using three-layer feedforward neural network. Any computer that can be connected to the Internet network can access services and operate various applications, which does not need to be installed and maintained, and has good scalability at the same time. Essay class, composition class, stores students' writing, and practice information are as shown in Table 2.

Through the update record function in the server behavior, the information is updated, the function of changing the student user data is realized, and the updated information is returned to the main page of the system. The control information of the next learning plan given systematically, such as controlling the teaching content, the difficulty of controlling the teaching process, and the next teaching strategy, aims to make the teaching content information be presented according to different students' levels and needs under the control of the system.

### 4.2. Analysis of Implicit Knowledge Base Based on Computer Network

The survey shows that about 79% of students believe that web-based assisted instruction is only some common learning aids such as ppt used by teachers during class, and their understanding of web-based assisted instruction is too narrow. However, many autonomous learning modules for college English viewing, listening, and speaking are poorly understood. In the implicit knowledge base, the sufficiency measure and necessity measure are defined as the form of possibility ratio. Domain experts do not need to provide accurate probability estimation, but only provide possibility ratio. The process of developing a self-learning implicit knowledge base in a network environment is summarized according to the general rules of software design and development education.

**Figure 6:**
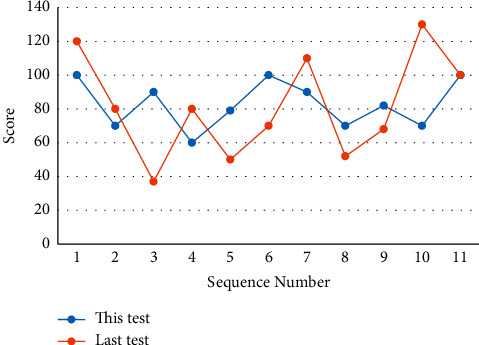
Data statistics interface.

First, according to the knowledge provided by the domain experts, the English knowledge was divided into 20 knowledge points, the English experts added questions for these 20 knowledge points as the test bank of the sample database, and the questions were selected to be representative. According to the characteristics of online learning, the content of each teaching unit and the logical relationship between the units were designed and scripted according to the constructivist learning theory. Therefore, importing formatted files allows the system administrator to manage the system user information in bulk, and the system administrator can update the user information in the system by uploading files with the user information or export the user information in the system to files. The system can record the last 20 test scores of students, and the statistical interface is shown in Figure 6.

**Figure 7:**
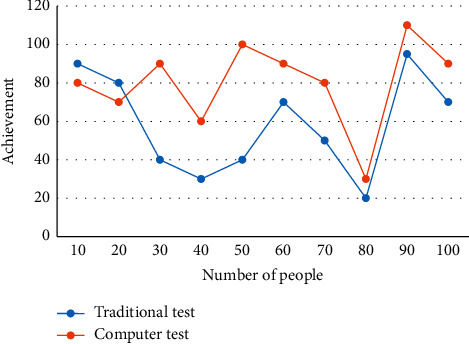
Comparison between paper-and-pen test and computer online evaluation.

Second, training and test samples are selected from this library, and when the network is well trained, students take questions from it for self-testing. Since the information on the Internet is mixed, students can easily devote their time and energy to information that is not related to the learning content. Therefore, teachers should provide online self-study for students, recognize the content, provide effective guidance on learning strategies, etc., and check the effectiveness of students' self-study. Through the interconnection of special equipment, it realizes the sharing of different types of educational resources files within the campus network segment, ensures the security, unity, and scalability of the educational resources file transfer system, and improves the management-level resources of various types of educational resources. The core is the function that can be used to determine whether data exist on a socket or whether data can be written. A comparative analysis method was used to experimentally compare traditional paper-and-pencil tests with computerized online assessments and analyze the impact of each, and the comparative results are shown in Figure 7.

**Figure 8:**
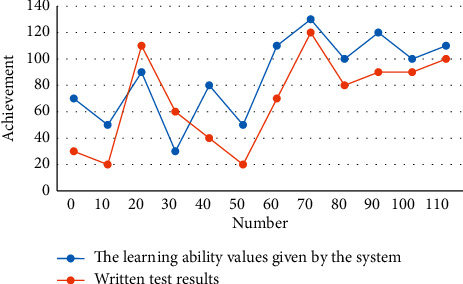
Comparison between the learning ability value given by the system and the written test score.

The results show that both groups basically meet the requirements of the syllabus, but the effect of system controlled learning in the computer test group is better than that in the traditional test. However, 81% of students prefer computer evaluation. The comparison between the learning ability value given by the system and the written test score is shown in Figure 8.

Therefore, the learning ability value given by the system is basically consistent with the final test result, which shows that the student model established by the system is basically reasonable.

Finally, the set of weighted coefficients and the set of thresholds are obtained after the conversion of the topics and rule tables provided by the domain experts to the learning in the form of internal codes, and the knowledge base stores the knowledge about the network structure, the composition of the weights, etc. There are database and its application system to store data efficiently and to meet user information requirements and processing requirements. This knowledge base can effectively prevent the application from being in blocking mode or entering a blocking state when the socket is in blocking mode. After the student selects a course and enters the diagnostic test phase, the system shall give the student the competency test questions for that level according to the level selected. Upon completion of the submission, the system should provide timely feedback and evaluation, and suggest appropriate adjustments to the level selected by the student.

## 5. Conclusion

Independent learning in the network environment is the new development direction of lifelong learning, and it gradually develops into an excellent educational environment parallel to the classroom. How to integrate different types of computer teaching resources into English classroom education, realize the sharing of high-quality English teaching resources, and stimulate students' interest in learning is the perfect education for the four major abilities of listening. The integration of oral, reading, and writing in English education and the change of traditional classroom teaching methods are the main goals of the current reform of English education in universities. In this paper, we analyze the problems of the current educational status and the limitations of the existing system by combining the current situation of teaching and research of domestic and foreign auxiliary education systems with the background of actual computer-based teaching and self-learning English based on computer networks supporting teaching methods. The article combines the practical application requirements, a detailed analysis of the system through the implicit knowledge base based on BP neural networks and computer networks, and the techniques of expert systems, the main construction steps, and the learning model of self-study knowledge representation of English. The teacher of computer-assisted design English teaching can fully utilize the technology of computers to create real-life scenarios for students in this authentic language learning environment, where students can perform semantic construction of knowledge in the classroom with bilateral stimulation of both visual and audio.

## Figures and Tables

**Table 1:** Test record.

Listing	Student-ID	Last time	Count	Grade
Type	Int	Data time	Int	Int
Can it be blank	N	N	N	N
Meaning	Student	Last test time	Number of tests	Total test score

**Table 2:** Data storage design—essay composition classes and attributes.

Attribute name	Title	Status	Creator
Attribute type	String	String	User
Explain	Title, null allowed	Writing state	Writing students

## Data Availability

The dataset can be accessed upon request.
